# An Auditory BCI System for Assisting CRS-R Behavioral Assessment in Patients with Disorders of Consciousness

**DOI:** 10.1038/srep32917

**Published:** 2016-09-13

**Authors:** Jun Xiao, Qiuyou Xie, Yanbin He, Tianyou Yu, Shenglin Lu, Ningmeng Huang, Ronghao Yu, Yuanqing Li

**Affiliations:** 1 Center for Brain Computer Interfaces and Brain Information Processing, South China University of Technology, Guangzhou, 510640, China; 2 Coma Research Group, Centre for Hyperbaric Oxygen and Neurorehabilitation, General Hospital of Guangzhou Military Command of People’s Liberation Army, Guangzhou 510010, China

## Abstract

The Coma Recovery Scale-Revised (CRS-R) is a consistent and sensitive behavioral assessment standard for disorders of consciousness (DOC) patients. However, the CRS-R has limitations due to its dependence on behavioral markers, which has led to a high rate of misdiagnosis. Brain-computer interfaces (BCIs), which directly detect brain activities without any behavioral expression, can be used to evaluate a patient’s state. In this study, we explored the application of BCIs in assisting CRS-R assessments of DOC patients. Specifically, an auditory passive EEG-based BCI system with an oddball paradigm was proposed to facilitate the evaluation of one item of the auditory function scale in the CRS-R – the auditory startle. The results obtained from five healthy subjects validated the efficacy of the BCI system. Nineteen DOC patients participated in the CRS-R and BCI assessments, of which three patients exhibited no responses in the CRS-R assessment but were responsive to auditory startle in the BCI assessment. These results revealed that a proportion of DOC patients who have no behavioral responses in the CRS-R assessment can generate neural responses, which can be detected by our BCI system. Therefore, the proposed BCI may provide more sensitive results than the CRS-R and thus assist CRS-R behavioral assessments.

Several behavioral assessment methods have been designed for assessments of brain-damaged patients suffering from disorders of consciousness (DOC), which generally include coma, vegetative state (VS), minimally conscious state (MCS) and emergence from MCS (EMCS). Among these methods, the Glasgow Coma Scale (GCS), the Coma Recovery Scale-Revised (CRS-R), the Full Outline of UnResponsiveness (FOUR), and the Wessex Head Injury Matrix (WHIM) are most widely applied methods in clinical assessments. Because an accurate evaluation of the level of consciousness in DOC patients is important for their diagnosis, their prognosis, and the monitoring of their treatment and rehabilitation effects, many researchers have studied these behavioral scales and suggest that the CRS-R is reliable and appears to be capable of distinguishing patients in MCS from those in VS^1^.

The Coma Recovery Scale (CRS) was initially proposed by Giacino *et al*. in 1991, and the revised version was more fully characterized in 2004^2^. The CRS-R consists of 6 subscales that address auditory, visual, motor, oromotor, communication and arousal processes. Each subscale contains several items, and there are a total of 23 hierarchically arranged items. For instance, the auditory subscale includes four items, and their corresponding item scores are 1, 2, 3, and 4 for auditory startle, localization to sound, reproducible movement to command, and consistent movement to command, respectively. Scoring is based on the presence or absence of specific behavioral responses to sensory stimuli administered in a standardized manner. Specifically, the scoring is performed from the item with the highest score to the item with the lowest score. The lowest item of each subscale represents reflexive activity, whereas the highest item represents cognitively mediated behaviors^3^. Examiners can reliably conduct the CRS-R with repeated measurements yielding stable estimates of a patient’s status. However, as a behavioral assessment method, the CRS-R has limitations^4^. First, it is difficult for DOC patients to sustain a long-term stable state to undergo the evaluation because of fluctuations in arousal level, fatigue, pain, and other factors. Second, the presence of motor impairment and tracheotomy may affect the behavioral responses and result in imprecise evaluation. For instance, several items of the CRS-R rely on the head nods/shakes (e.g., communication subscale) or repetitive word-like sound (e.g., oromotor/verbal function subscale) for scoring. This task is not easy or even impossible for most patients with tracheotomies to perform. Moreover, there are problems of inter-rater reliability (the reproducibility of CRS-R scores between different raters) and test-retest reliability, which are related to the examiners and the states of the patients, respectively. The inter-rater reliability for CRS-R scores may be evaluated using an index of κappa values. κappa values of 0.5–0.8 indicate moderate-to-good inter-rater reliability^2^,^5^,^6^,^7^. These limitations of the CRS-R behavioral assessment can yield a high rate of misdiagnosis in DOC patients as reported in recent studies. It was observed that 37% to 43% of patients diagnosed as being in a VS actually exhibited signs of awareness^1^,^8^,^9^,^10^.

Considering the aforementioned limitations, brain-computer interfaces (BCIs) may represent a potential solution, because they can directly detect endogenous brain responses to external stimuli without the need for any behavioral or verbal expression. In recent years, several BCI systems have been successfully applied in awareness detection and environmental communication for DOC patients^11^,^12^,^13^,^14^,^15^. In [11], a hybrid BCI that combines visual P300 and steady-state visually evoked potentials (SSVEPs) was developed to detect awareness in DOC patients. The patient was instructed to look at his/her own photo or another unfamiliar photo displayed on a computer screen according to different experimental cues. Awareness was then assessed by analyzing whether the command was correctly followed. Cruse *et al*. investigated the ability of DOC patients to perform demanding motor imagery (MI) tasks using a MI-based BCI system. Sixteen behaviorally VS/MCS patients were instructed to imagine either squeezing their right hand or moving all their toes. Their results indicated that 19% of the patients were able to accurately perform the tasks, with cross-validated accuracies between 61% and 78%^13^. Wang *et al*. proposed an audiovisual BCI using congruent audiovisual number stimuli to detect awareness in DOC patients. Seven DOC patients were included in the experiment and were asked to selectively attend to the target numbers cued by the instructions. Five patients exhibited command following as well as number recognition^15^.

Some DOC patients may lose the ability to fixate their gaze, which is generally necessary for the use of visual BCIs^11^,^16^. Therefore, auditory BCI approaches based on various levels of conscious have been applied to DOC patients^17^,^18^,^19^. For instance, Real *et al*. employed an auditory oddball paradigm involving two short tones to differentiate between VS and MCS in 45 DOC patients. The results indicated that the prevalence of P300 was significantly higher in healthy subjects than in the patients but did not reveal a significant difference between VS and MCS patients^17^. Lulé *et al*. presented a four-choice auditory P300 BCI with spoken words “yes”, “no”, “stop”, and “go”^12^. Among the 18 DOC patients involved, one MCS patient exhibited command following with an offline accuracy of 57% for the BCI experiment. Furthermore, one lock-in syndrome (LIS) patient achieved a significant online correct rate of 60% and was able to communicate with the BCI. The other patients did not exhibit any response to commands and could not communicate through the BCI. However, to our knowledge, BCI studies with regard to assisting traditional assessments for DOC patients based on behavioral scales such as the CRS-R have not been reported. Specifically, the CRS-R includes 23 items, such as auditory startle, that were not considered in the existing BCI paradigms designed for DOC patients. Therefore, these BCIs cannot be directly used to assist CRS-R, for example, whether a DOC patient is responsive to auditory startle or not cannot be evaluated using these BCIs based on command-following paradigms. Furthermore, the performance of these BCIs designed for DOC patients is generally poor, which is mainly because recognition levels, which often play an important role in a BCI system, substantially differ between healthy subjects and DOC patients. Therefore, it has been challenging to design effective BCI systems for these patients.

In this study, we explored the application of BCIs in assisting CRS-R behavioral assessments, with a focus on the auditory startle item in the auditory subscale. In the CRS-R assessment, eyelid flutter or blinking is considered a behavioral response to an auditory startle, but it is difficult to estimate how long to wait, and there is a risk of spontaneous blinking being interpreted as a volitional response^20^. Eye blinks are typically classified into three categories: spontaneous eye blinking that occurs frequently, reflexive eye blinking evoked by an external stimulus, and voluntary eye blinking due to intentional eye closing^21^. However, it is difficult for the examiner to distinguish such tiny behavioral differences without systematically recording eye blinks under different conditions. Therefore, the assessment of responsiveness to auditory startle based on eyelid flutter or blink may be one of the factors that lead to the high rate of misdiagnosis in DOC patients. We thus developed an auditory BCI with an oddball paradigm to assist the assessment of responsiveness to auditory startle. For instance, when a DOC patient does not exhibit any behavioral response to auditory startle in the CRS-R assessment, the corresponding item score is 0. In this case, if the patient achieves a significant accuracy rate in the BCI measurement, then the score of 0 in the auditory subscale assessment can be revised to be 1. Most of the previously mentioned BCI paradigms have been designed in a supervised manner; i.e., an initial model was first constructed based on a dataset collected in the training session, which was then used to classify the incoming signals and provide online feedback. This model training process may increase the difficulty of applying BCI approaches for DOC patients, as the arousal period of DOC patients is usually too short to obtain a reliable model. Therefore, a new peak detection algorithm was used in our BCI system, and the initial training session was not necessary. During the experiment, standard and deviant stimuli were randomly presented to the subjects. It was believed that mismatch negativity (MMN) and P300 event-related potentials would be elicited and could be reliably detected by the BCI system. The detection result was presented as online feedback, and the clinical examiner could make judgments based on the results. The efficacy of our auditory BCI with the peak detection algorithm was first validated by the results of five healthy subjects. Next, nineteen DOC patients participated in our experiments involving both the BCI and CRS-R assessments. Comprehensive data analysis demonstrated the effectiveness of our BCI system in detecting responsiveness to auditory startle. The results of the BCI assessment were used to polish up the item score for auditory startle in CRS-R. The auditory BCI system may provide a more objective and sensitive way to evaluate auditory startle and can be used to assist CRS-R behavioral assessments.

## Results

### Healthy Subjects

The average online accuracy of the five subjects was 84.6 ± 7.8%, and each subject achieved an online accuracy greater than the significance level of 36.7% (*p* < 0.001, χ^2^-test). This result validated the facts that the evoked responses were sufficient to provide online feedback, and that the peak detection algorithm was effective for detecting the electroencephalography (EEG) response to auditory startle.

**Figure 1:**
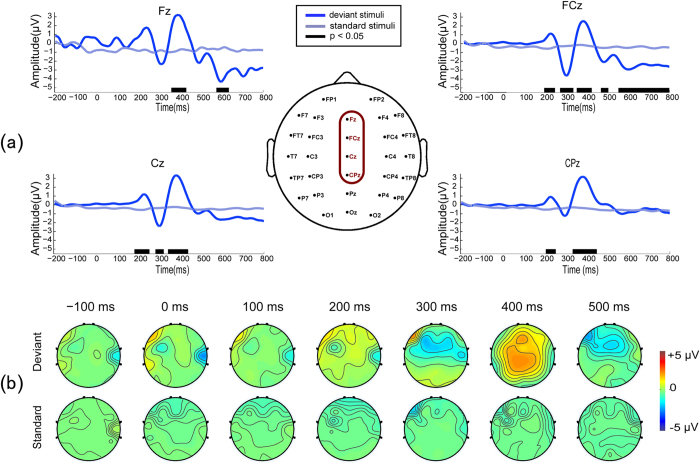
ERP results for the five healthy subjects. (**A**) The overall average ERP waveforms (from 200 ms pre-stimulus to 800 ms post-stimulus) in the selected channels (“Fz”, “FCz”, “Cz”, and “CPz”) and the time intervals with significant differences of the waveforms elicited by the deviant and standard stimuli (point-wise running *t*-test with a cluster size of 7 for multiple comparison, *p* < 0.05, indicated by the black bars). The blue and gray lines denote the waveforms evoked by the deviant and standard stimuli, respectively. (**B**) The averaged scalp maps of all five healthy subjects. The blue areas reveal that negative responses (MMN component) occurred at approximately 300 ms, mainly in the frontal-central region, and the red areas indicate that the positive responses (P300) achieved a maximum at approximately 400 ms and were distributed in the frontal-central and central regions.

To further demonstrate the efficacy and reasonability of our peak detection algorithm, we performed an offline ERP analysis. The averaged waveforms and scalp topographies for the healthy subjects are presented in Fig. 1. The results reveal that the MMN elicited by the deviant stimulus occurred slightly more than 300 ms after stimulus onset, and P300 occurred at approximately 400 ms after onset, following the MMN. The MMN position in this study was delayed than in the previously reports of Näätänens *et al*.^22^. The longer latency of the MMN in our experiment might be a result of the experimental stimuli (the recorded sound of a clap)^23^. Furthermore, amplitude variation was rarely observed for the standard stimuli, and this finding is consistent with the physiological principles of MMN and P300 presented in ref. 24. We also performed point-wise running *t*-tests to compare the differences between target and non-target responses, where a cluster size of 7 was used for a multiple comparison correction^25^,^26^,^27^. From Fig. 1(A), we can see that there were remarkable differences in the period of 250–500 ms between the responses elicited by the deviant and standard stimuli. Additionally, the averaged scalp maps shown in Fig. 1(B) revealed that the negative maximum locations were distributed mainly in the frontal-central region, including the electrodes of “Fz” and “FCz”. The positive maximum locations were distributed mainly in the frontal-central and central regions, including the electrodes of “FCz”, “Cz” and “CPz”. These results further confirmed that the auditory startle could elicit discriminable brain responses in physiologically reasonable brain areas. Finally, the selected time window of 250–500 ms was reasonable and reliable for performing peak detection.

### Patients with DOCs

The high online accuracy rates, the apparent ERP waveforms and the reasonable distribution in scalp maps for the healthy subjects demonstrated the effectiveness of the experimental paradigm and the peak detection algorithm. We further performed online tests for 19 DOC patients over approximately seven months, using the auditory passive BCI system with the same parameters as those for healthy subjects. The CRS-R-based behavioral results and BCI-based online results were obtained for these patients, as detailed in Table 1. Note that patients 4 and 11 performed two BCI sessions because their CRS-R evaluations changed from VS to MCS. For most of the patients, the results based on the BCI assessment were consistent with those based on the CRS-R. Specifically, for both assessment methods, 14 of the 19 patients, including patients 1, 2, 3, 6, 8, 9, 10, 11, 14, 15, 16, 17, 18, and 19, were classified into the responsive group (a significant group average for the online accuracy, i.e., 0.72 ± 0.19, was achieved); meanwhile, patients 5 and 7 were classified into the nonresponsive group by both the BCI and CRS-R assessments. Interestingly, the remaining 3 patients were classified into the inconsistent group. Patients 12 and 13 did not exhibit any behavioral response during CRS-R assessment, and patient 4 did not exhibit any behavioral response during the first CRS-R assessment but exhibited a behavioral response during the second CRS-R assessment. These three patients were suggested to be responsive to auditory startle by BCI system the corresponding item score was suggested to be 1.

**Figure 2:**
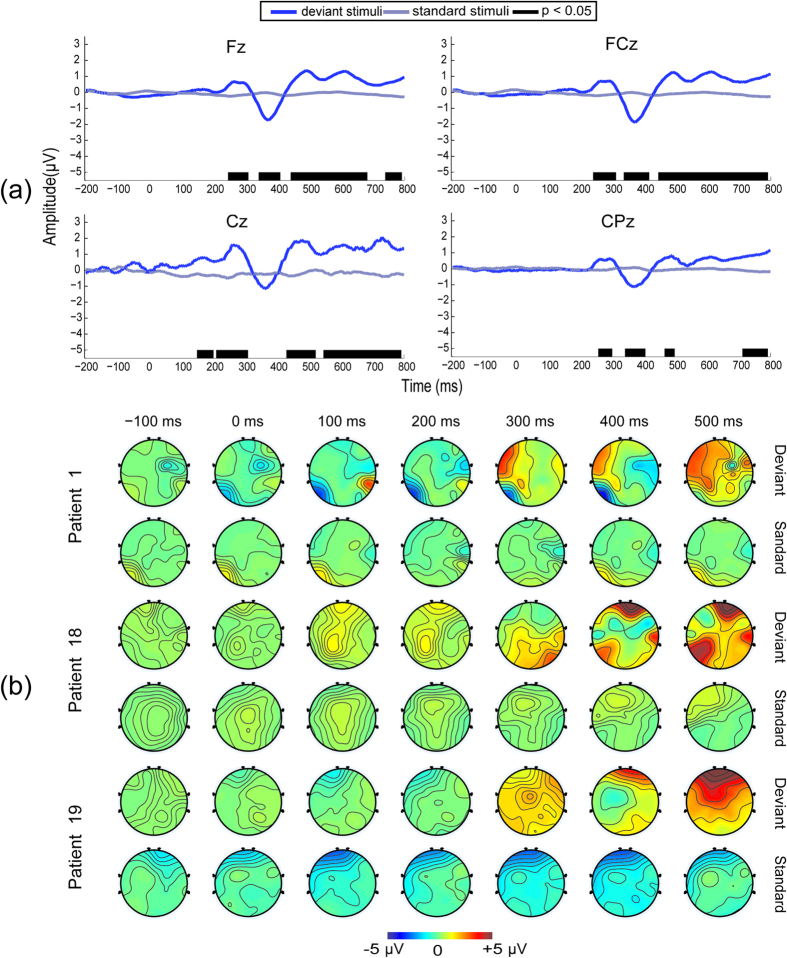
ERP results for the 14 responsive patients. (**A**) The overall group-average ERP waveforms (from 200 ms pre-stimulus to 800 ms post-stimulus) from the four selected channels “Fz”, “FCz”, “Cz”, and “CPz”. The blue lines represent the average waveforms elicited by the deviant stimuli, and the gray lines represent those elicited by the standard stimuli. The black bars at the bottom of each sub-figure indicate the time intervals with significant differences between the deviant and standard stimulus responses using point-wise running *t*-tests for each electrode, where a cluster size of 7 was used for multiple comparison (*p* < 0.05). (**B**) Scalp topographies of representative patients 1, 18, 19. For patients 1 and 18, the MMN response occurred at approximately 400 ms (mainly in the frontal-central region, e.g., “Fz” and “FCz”), and P300 occurred at approximately 500 ms (distributed in the frontal-central and central regions, including the electrodes of “FCz”,“Cz” and “CPz”). For patient 19, there was an early positive response at 300 ms, and the MMN and P300 then occurred at approximately 400 and 500 ms, respectively, with the same distribution as in the other two patients.

To further illustrate the reasonability of our BCI detection results, we also performed an offline ERP analysis using the data from the DOC patients. For the 14 patients who were judged to be responsive by both BCI and CRS-R assessments, Fig. 2(A) presents the overall group-average ERP waveforms from the four selected channels (“Fz”, “FCz”, “Cz”, and “CPz”) and indicates the time intervals with significant differences between the responses to the deviant stimuli and those to standard stimuli. The overall group-average ERP waveforms were obtained by averaging the baseline-corrected EEG epochs across 20 trials first and then over all 14 patients. As can be observed from Fig. 2(A), a negative peak (MMN) emerged within 300–400 ms (at “Fz”, “FCz”, and “CPz”), which was followed by a positive peak (P300) within 400–500 ms (at “Fz”, “FCz”, and “Cz”). The results for these patients were partially consistent with those for the healthy subjects in terms of the related electrode locations (the frontal- and central- areas). Compared with the healthy subjects’ averaged ERPs, there was a time delay of approximately 50 ms (Table 2 and Fig. 2) in the responsive group-averaged ERPs. Furthermore, the late negativity of approximately 600 ms observed in healthy subjects was not visible in these responsive patients. For the representative patients 1, 18 and 19, scalp topographies of their EEG responses to the two types of stimuli (deviant and standard) are presented in Fig. 2(B). These results indicate that the frontal-central regions, e.g., “Fz” and “FCz”, were associated with an MMN in the range of 300–400 ms, and the P300 was distributed mainly in the frontal-central and central regions, e.g., “FCz”,“Cz” and “CPz”, occurring in the range of 400–500 ms. These findings were also consistent with the neurophysiology results reported in previous publications^24^.

**Figure 3:**
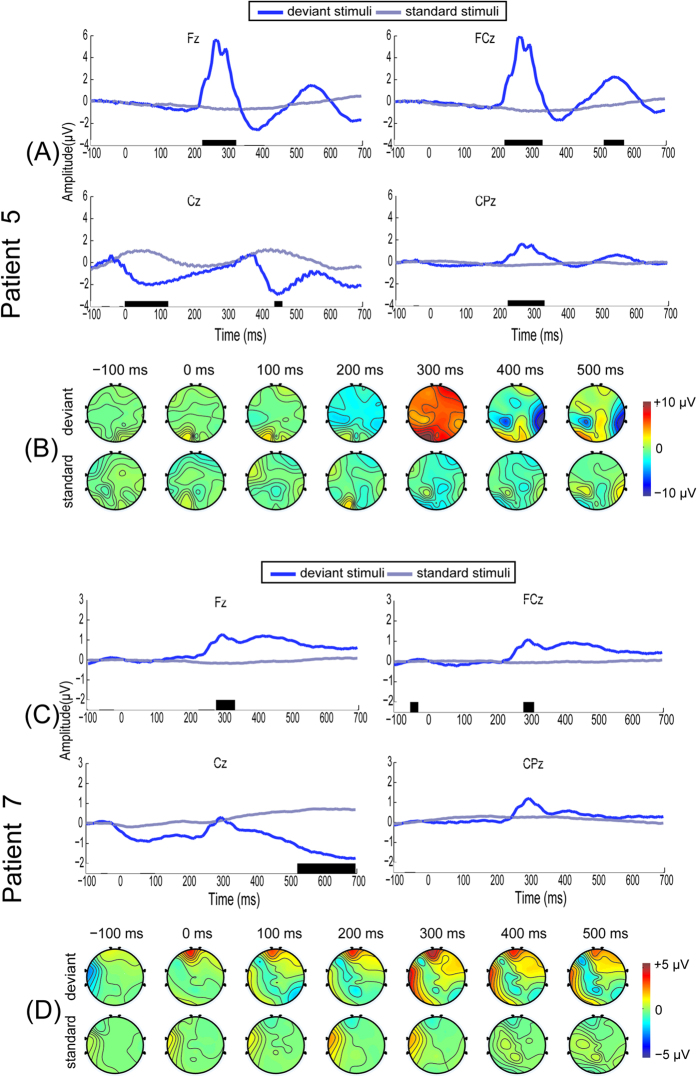
ERP results for patients 5 and 7. (**A**) ERP waveforms for patient 5. A large positive peak occurred between 200 and 350 ms in channels of “Fz” and “FCz”. The black bars indicated that the time intervals with significant differences betweeen the waveforms elicited by the deviant and standard stimuli were also between 200 and 350 ms in “Fz” and “FCz”. (**B**) Scalp topographies of patient 5. Remarkable positive responses were distributed in the whole scalp at approximately 300 ms. (**C**) ERP waveforms for patient 7. There was a tiny positive peak at approximately 300 ms for all of the selected channels. The time intervals with significance were approximately 300 ms in the channels “Fz” and “FCz”. (**D**) Scalp topographies of patient 7, which indicate that the EEG changed slowly during the time window from 200 to 500 ms.

Moreover, the two nonresponsive patients (patients 5 and 7, verified by both BCI and CRS-R assessments) exhibited very different results in terms of their ERP waveforms and scalp topographies. Patient 5 exhibited a remarkable early positive peak at 250 ms but no MMN component in the ERP waveforms for the deviant stimuli, as shown in Fig. 3(A). Because the positive peak was not in the predefined time window, it was not successfully detected by the peak detection algorithm. This patient’s topographical results revealed that the major signal changes were distributed over the whole scalp at approximately 300 ms, Fig. 3(B). Patient 7 exhibited a tiny positive peak occurring at approximately 300 ms, but no MMN was observed in the ERP waveforms for the deviant stimuli, as shown in Fig. 3(C). This patient’s scalp topographies are shown in Fig. 3(D) and indicated that the EEG changed slowly during the time window from 200 to 500 ms. Overall, for patients 5 and 7, there were no MMN and P300 components observed in their ERP waveforms.

**Figure 4:**
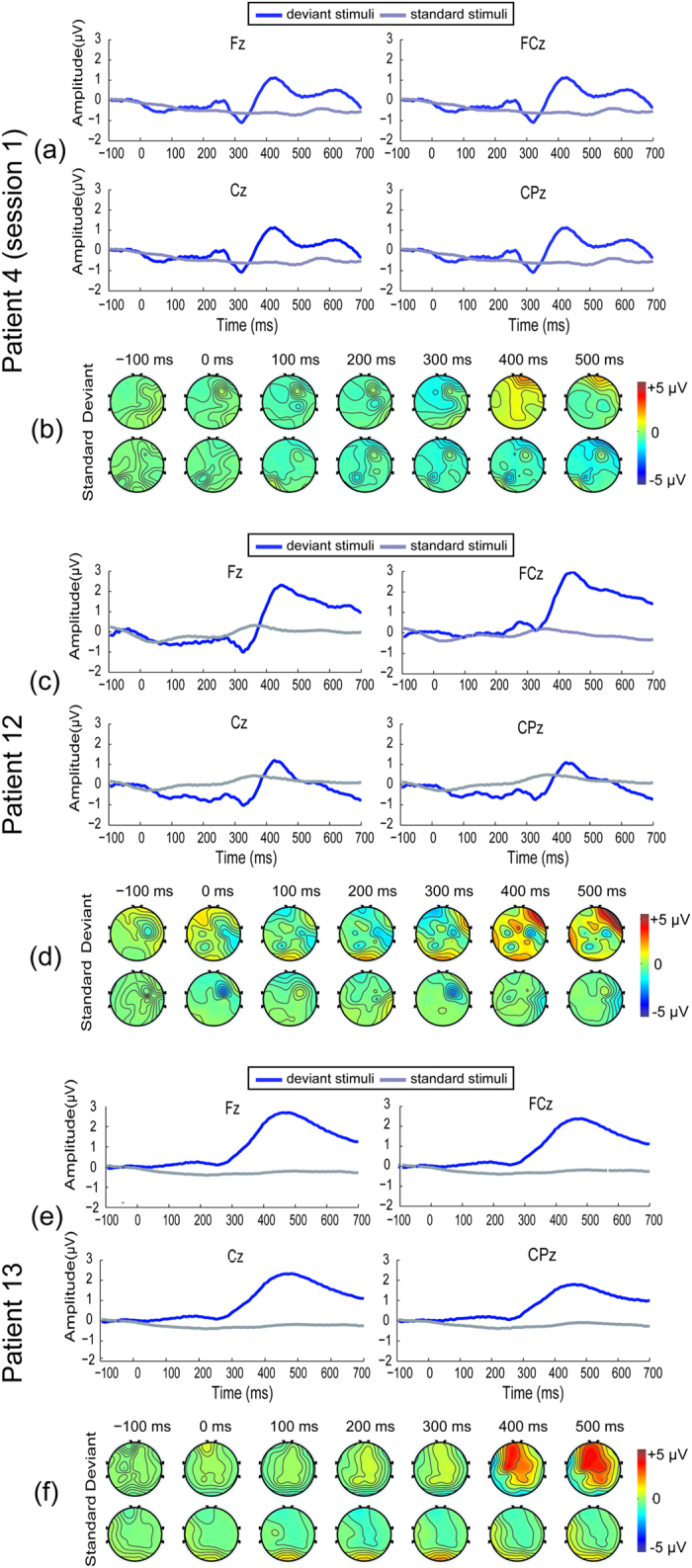
ERP results for patients 4, 12 and 13. (**A**) Patient 4’s ERP waveforms from the first BCI session (no behavioral response observed at that time). The negative and positive peaks corresponding to the time periods and channel locations were similar to those in the responsive group, as judged by both the CRS-R and BCI assessments. (**B**) Scalp topographies of patient 4 in the first BCI session. (**C**) Patient 12’s ERP waveforms, MMN and P300, could be observed in the range of 300–500 ms at “Fz”, “FCz”, “Cz”, and “CPz”. (**D**) Scalp topographies of patient 12. (**E**) Patient 13’s ERP waveforms. There was a broad positive peak from 400 to 500 ms but no MMN components. (**F**) Scalp topographies of patient 13, which indicate that the positive response was focused in the middle of the brain.

The three VS patients (4, 12 and 13) showed no behavioral response in the CRS-R measurement but achieved significant online accuracy in the BCI measurement (see Table 1). Note that patient 4 did not show any behavioral response in the first CRS-R-based measurement but did in the second CRS-R-based measurement. This difference may be due to his recovery from VS to MCS during the period of our experiment. For patients 4 and 12, the ERP waveforms indicated the MMN in the time interval of 300–350 ms and the following P300 at approximately 400 ms (see Fig. 4(A) for patient 4 and Fig. 4(C) for patient 12). Furthermore, the scalp topographies of the EEG responses to the two types of stimuli (deviant and standard) are presented in Fig. 4(B) (patient 4) and Fig. 4(D) (patient 12). These results indicated that the frontal-central sites, i.e., “Fz” and “FCz”, were associated with the MMN, and the frontal-central and central regions were associated with the P300. Overall, for patients 4 and 12, we obtained ERP results similar to those of the responsive group, as judged by both the CRS-R and BCI assessments. For patient 13, we did not observe the MMN; however, the P300 appeared in the time interval 400–500 ms (Fig. 4(E)). The topographies of the EEG responses indicated that the frontal and central regions were associated with the P300 (Fig. 4(F)). Note that these results do not imply that the peak detection algorithm could detect the target only when the both MMN and P300 were simultaneously present for a patient. If one of MMN and P300 appeared, the difference between the maximum and minimum in the related time window could be sufficiently large. In this case, the algorithm was still able to detect the target.

The amplitudes and latencies of the MMN and P300 components were calculated for each of the 5 healthy subjects and 17 patients; the two patients who did not show MMN and P300 were excluded. For each component, we chose the maximum amplitude and determined its associated latency^28^. The mean amplitudes and peak latencies of MMN (FCz) and P300 (Cz) for each group are presented in Table 2. The results of a *t*-test indicated that there was no significant difference between the healthy subjects and responsive patients in both MMN amplitudes (*p* > *0.05*) and P300 amplitudes (*p* > *0.05*). However, a significant difference was found in terms of the latencies of MMN (*p* < *0.01*) and P300 (*p* < *0.05*) between the two groups. Specifically, the latencies of MMN/P300 were significantly longer for the responsive patients than for the healthy subjects, which are consistent with the results reported in previous work^29^,^30^. In addition, there was a broad late positive component between 500 and 800 ms in the ERPs of the responsive DOC patients that resembled the late positive component (LPC) associated with the subject’s voluntary mental task of discriminating the stimulus properties, as reported in previous studies^31^,^32^. The reason for the lack of a comparison with the inconsistent group is that the number of samples did not attain statistical significance^33^.

## Discussion

Recently, researchers have used the “command following” BCI paradigm to detect awareness of DOC patients^11^,^12^,^14^. It is believed that if a patient is able to follow an instruction to imagine his/her left/right-hand movement or gaze at different visual stimuli, then he/she is conscious. This method could help to reduce the possibility of misdiagnosis of those DOC patients who have difficulty exhibiting any behavioral response to external stimuli. However, this type of method was not designed to objectively and systematically record subtle changes in each individual function for DOC patients, which is often performed using the CRS-R. The CRS-R assessment has limitations in differentiating between MCS and VS because voluntary behaviors are often regarded as a sign of an MCS state, but voluntary and reflexive behaviors are difficult to distinguish, and the subtle signs of voluntary movements may be missed^4^,^20^. The main objective of our study was to investigate the feasibility of using BCI systems to assist the CRS-R-based behavioral assessments. Specifically, we proposed an oddball auditory BCI system to assist in the evaluation of auditory startle in the CRS-R rather than to replace it. This BCI system was able to provide real-time feedback, which was calculated by a peak detection algorithm based on the time-locked features of MMN and P300 elicited by the deviant stimuli. The combination of CRS-R and BCI assessments may help to obtain more precise, objective and consistent diagnoses. In this manner, misdiagnosis caused by habituated eye blinking or missed subtle actions might be partially avoided with the BCI system because brain responses elicited by the auditory stimuli can be directly detected.

The MMN is generated by the brain’s automatic response to a physical infrequent stimulus that deviates from the preceding frequent stimulus in repetitive auditory inputs, and it is typically observed as a frontal-central negativity of approximately 0.5–5 μV in amplitude that occurs with a latency of 250 ms. The P300 is a large, broad, positive component with typical peak latency between 300 and 400 ms after the onset of a rare, task-relevant stimulus^22^. Both events can be elicited by a carefully designed auditory oddball paradigm. In this study, the efficacy of the peak detection algorithm was first verified using experimental data from healthy subjects. For these healthy subjects, the overall average ERP waveform showed that the MMN appeared at 300 ms and the P300 presented in the next 100 ms after the onset of the stimuli. This timing validated our selection of a fixed time window from 250 to 500 ms for the online peak detection algorithm. Together, scalp maps confirmed that the electrodes related to the MMN and the P300 were distributed over the regions of the frontal and central areas, which is consistent with the existing results^24^,^34^. These results demonstrated the effectiveness of channel selection in our BCI algorithm. For 16 of the 19 patients involved in our experiment, the BCI assessment results were consistent with those from the CRS-R assessment, also demonstrating the effectiveness of our peak detection algorithm for DOC patients. The peak detection algorithm is promising for DOC patients. Furthermore, the proposed peak detection algorithm relies on and emphasizes the presence of MMN or P300. Thus, the positive results might be neurologically reasonable.

By tracking these three patients (patients 4, 12, and 13) who were behaviorally nonresponsive but were judged to be responsive by the BCI assessment, we found that patient 4 exhibited a behavioral response to auditory startle a few days after the experiment (we immediately conducted the second BCI experiment for this patient, and he still exhibited significant EEG responses). Patient 12 exhibited a behavioral response to auditory startle after discharge from the hospital. Because patients 12 and 13 were consistently behaviorally nonresponsive in the hospital, they were further evaluated based on brain-stem auditory evoked potentials (BAEPs), which are usually used to test auditory sensitivity and the neurological status of the auditory brain stem pathway. The BAEP results revealed that patients 12 and 13 had completed waveforms I, II, III, IV and V, thus implying that their brain stems function was intact^35^.

The two BCI-nonresponsive and CRS-R-nonresponsive subjects, i.e., patients 5 and 7, did not exhibit any MMN or P300 components. Patient 5 was a NTBI patient. For this patient, only a positive waveform in the range of 200–300 ms was observed over the whole scalp, and no negative peak was presented. The ERP pattern led to a failure of the peak detection algorithm to distinguish the target and non-target responses. For patient 7, EEG differences between the deviant and standard stimuli were barely observed. It has not been confirmed that the appearance of MMN or P300 was a necessary condition for the auditory startle. Therefore, the negative results for patients 5 and 7 based on the BCI assessment did not necessarily mean that these patients were non-responsive to auditory startle. However, these two patients did not exhibit any behavioral responses to auditory startle even after discharge from the hospital.

In summary, among the 19 patients involved, all 14 patients who were responsive to CRS-R were detected by our BCI system. Furthermore, three patients who were behaviorally nonresponsive according to the CRS-R assessment were judged to be responsive by the BCI. The last two patients in the nonresponsive group required further confirmation or long-term tracking. Therefore, we conclude that the BCI assessment might be more sensitive than the CRS-R-based behavioral assessment. The reliability of BCI assessment was estimated primarily by combining the results of the CRS-R behavioral assessment, the analysis of the ERP and tracking the progress of patients’ recovery.

As mentioned in the introduction, a very small proportion of DOC patients are able to communicate using an auditory or MI-based BCI system. Those BCI systems require patients to understand instructions, and then follow commands to count the target stimuli in an auditory sequence or perform motor imagery tasks. The aforementioned BCI systems detected the covert consciousness of DOC patients using a command-following task. Unlike the BCI-based consciousness detection, no instruction was used in the experiment. We assessed the patients’ response to auditory startle, one of the lowest items in the CRS-R. This response represented reflexive activity. Therefore, language understanding ability is not required of the patients, which may be similar to the design of the auditory startle item in the CRS-R. These facts might have resulted in the relatively high percentage of patients who achieved significant BCI accuracies. Furthermore, the results yielded by the BCI algorithm might be useful for overcoming the subjective bias that often occurs in the CRS-R assessments and reducing the rate of misdiagnosis.

Accurate evaluation of the DOC patients is of major importance for their daily management. Our BCI system is primarily used for assisting diagnosis. There are four possible cases. (i) If the DOC patient is responsive in both the CRS-R and BCI assessments, the item score is 1. (ii) If a reliable and consistent behavioral response can be detected by CRS-R in a patient and the result of the BCI assessment is negative (miss), then the CRS-R behavioral result can be preferred because there are some people who are not able to use BCIs (BCI illiteracy). (iii) If the patient does not exhibit any behavioral response in a CRS-R assessment but achieves a significant accuracy rate in the BCI measurement, then the BCI assessment result can be used. (iv) If no behavioral response can be detected by CRS-R and the result of the BCI assessment is also negative, the item score is zero. This score implies that the patient is actually nonresponsive to auditory startle or can be responsive to auditory startle but both BCI and CRS-R missed it. We must improve the assessment methods to solve or alleviate the latter problem.

Because this was a pilot study, several improvements and extensions could be made. First, because the peak detection algorithm relies on use of a fixed time window and channels that were chosen based on the healthy subjects’ EEG data and previous studies^36^, the algorithm may fail for some special patients whose ERP patterns exhibit different temporal or spatial properties. Thus, optimizing the time window and channel setting may improve the detection performance of our algorithm. Further study regarding how to combine feature selection with the peak detection algorithm in which there are no labeled training data would be required. Second, the number of patients involved in our experiment was relatively small. Future studies should increase the number of patients included to improve the feasibility of our system in clinical applications. Third, an additional goal of our future work is to develop new BCI systems for other items of the CRS-R scale.

## Materials and Methods

### Subjects

We first conducted the experiment involving five healthy subjects without a history of neurological disease (four males; mean age ± SD, 29 ± 3 years) to validate our BCI system. Next, nineteen DOC patients (18 male, 1 female; aged 16–72 years) from the General Hospital of Guangzhou Military Command of People’s Liberation Army, participated in our experiments, with their history and status presented in Table 3. Two of the nineteen patients (patients 4 and 11) underwent a second BCI experiment at the time of detectable evolution from VS to MCS, according to the CRS-R assessment. The other seventeen patients participated in only one session of the BCI experiment.

The experiments for the healthy subjects and DOC patients were approved by the Ethical Committee of the General Hospital of Guangzhou Military Command of People’s Liberation Army, which complies with the Code of Ethics of the World Medical Association (Declaration of Helsinki), and written informed consent for the experiments and publication of their individual details in this manuscript was obtained from all of the healthy subjects and the patients’ legal surrogates.

### Data Acquisition

A SynAmps2 amplifier (NeuroscanCompumedics, USA) and a 32-channel EEG cap (LT37), which follows the standard 10–20 system, were used to record the scalp EEG signals at a sampling rate of 250 Hz with bandpass filtering between 0.05 and 100 Hz. An electrooculogram (EOG) was recorded from two pairs of electrodes (“HEOR” and “HEOL” and “VEOU” and “VEOL”) to remove ocular movement artifacts from the EEG signal. The impedances of all electrodes were kept below 5 kΩ.

### Experimental Paradigms

The oddball paradigm of our BCI system was designed to simulate the behavioral auditory startle evaluation in CRS-R, in which only a loud clapping sound was used as a stimulus. Specifically, the recorded sound of environment background noise (approximately 40 dB) and the recorded sound of a clap (approximately 90 dB) were randomly presented to subjects as the frequent standard and rare deviant stimuli respectively. These settings were used to keep the BCI assessment as close as possible to the CRS-R-based behavioral assessment. Additionally, MMN is more likely to be evoked in patients with brain injury in response to complex sounds rather than sinusoidal tones^37^. Therefore, a spectrally rich clapping sound, rather than a single-frequency tone, was used as the deviant stimulus in this study.

**Figure 5:**
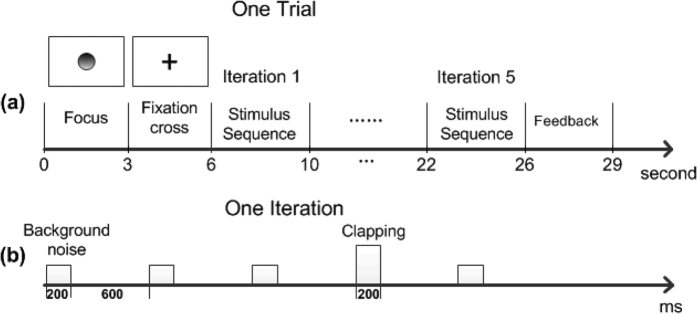
Timing of the auditory oddball paradigm for healthy subjects. (**A**) The procedure for one trial, including five repeated stimulus iterations (rounds). (**B**) The time course of one iteration in a trial. There were five stimuli in one iteration, namely, an infrequent deviant stimulus and four frequent standard stimuli, and these stimuli were presented in a random order. The duration of stimulus was 200 ms, and the inter-stimulus interval (ISI) was 600 ms.

The five healthy subjects first attended the experiment to validate our BCI system. Each healthy subject completed one session of 30 trials; one trial included five repeated stimulus iterations (rounds), as shown in Fig. 5(A). At the end of each trial, the peak detection algorithm was applied to the collected EEG data of this trial to determine the stimulus that evoked a brain response. Specifically, if the response to the deviant stimuli was successfully detected, then an online feedback of a tick was presented for 3 s. In contrast, there was no presentation on the screen for an incorrect result. In addition, the online ratio of the number of trials with correct detection to the total trial number was calculated and displayed on the bottom of the screen. The online feedback was mainly used by the examiners to evaluate the patient. One stimulus iteration consisted of five stimuli (Fig. 5(B)), one deviant and four standard (20% occurrence of rare stimuli), and these stimuli were presented in a random order and binaurally through in-ear headphones. The rare sounds were supposed to elicit MMN and P300 potentials that could be detected by the BCI system. A single trial for healthy subjects lasted for approximately 29 s.

Next, the BCI system was applied to the DOC patients. Two different auditory startle assessment methods were utilized and compared in this study: (1) CRS-R-based assessment and (2) BCI-based measurement. The CRS-R-based assessment was conducted by a clinician. Following the standard protocol, the clinician presented a loud clap directly above the patient’s head, i.e., out of his/her view, for 4 trials. If the eyelid flutter or blink occurred immediately following the stimulus onset in at least 2 trials, the patient received a score of 1 for the auditory startle item; otherwise, the patient received a score of 0.

The BCI-based measurement was conducted using the auditory oddball paradigm, as described above. Each BCI session consisted of 20 trials, with an inter-trial break of 1–10 s to ensure that the patient was in a stable state. The procedure for each trial was similar to that for healthy subjects except that one trial for the patients included 10 repeated stimulus iterations and lasted for approximately 49 s (refer to Fig. 5). Here, we used 10 iterations of stimuli for the patients to obtain a satisfactory detection performance because the brain patterns (MMN and P300) in the patients were generally weaker than those of the healthy subjects.

### EEG Data Analysis

#### Online Peak Detection Algorithm

We describe the peak detection algorithm based on the test for DOC patients, which classified EEG signals and provided online feedback. For each trial, the collected EEG data were processed using the following steps. (1) The raw EEG data were filtered with a sixth order minimum-phase FIR band-pass filter between 0.1 and 10 Hz and then were linearly detrended. Thereafter, ocular artifacts were reduced by applying a time-domain regression method with the EOG recording^38^. (2) We selected four channels (“Fz”, “FCz”, “Cz”, and “CPz”) beforehand for target detection according to related ref. 36. Using the filtered data from the selected channels, we constructed 50 data epochs corresponding to 50 stimuli (10 iterations of one deviant stimulus and four standard stimuli), where each epoch ranged from −200 to 800 ms relative to a stimulus-onset and corresponded to a data matrix (4 selected channels by 250 data points). The data epochs were averaged across repetitions for each stimulus, and five average data epoch matrices were obtained, each of which corresponded to a stimulus. (3) We chose a time window of 250–500 ms, which may contain the MMN and P300^24^,^29^,^36^,^39^. For each averaged data epoch matrix, the minimum value was found in the range of 250 to 400 ms, the maximum value was found in an interval of 100 ms following the minimum for each channel, and the difference between the maximum and the minimum was further computed. Therefore, for each of the four selected channels and each of the five stimuli, we obtained a difference value. A 4-dimensional difference vector was constructed for each stimulus by concatenating these difference values from all the selected channels, and a 4-by-5 difference matrix was obtained for each trial. (4) A voting method was used to determine the target stimulus. For each channel (a row of the difference matrix), the vote was given to the stimulus with the largest difference. The stimulus with the maximum number of votes was the target. Furthermore, if there were more than one stimulus that received the same maximum number of votes, we compared the difference values of these stimuli and determined the target stimulus with the maximum difference.

#### Statistical Analysis

The BCI accuracy, namely, the ratio between the number of trials with correct responses and the total number of presented trials, was calculated as a preliminary measure. To obtain an index of responsiveness to auditory startle, further statistical analysis of the accuracy was performed based on the χ^2^-square test. The χ^2^-square statistic was calculated as below^40^:


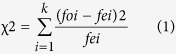


where *f*_*oi*_ and *f*_*ei*_ were the observed and expected frequencies of the *i* th class (*i* = 1, 2, ..*k*). In this study, there were two classes (hit and miss), i.e., *k* = 2. A hit was counted if the output class in a trial was deviant stimulus (a true positive); otherwise, a miss was recorded (a false positive). There were five choices in our paradigm, namely, one deviant stimulus and four standard stimuli. The chance level for choosing the deviant stimulus (hit) was 20%, whereas the chance level for choosing the standard stimuli (miss) was 80%. *f*_*o*1_ and *f*_*o*2_ were the observed numbers of hits and misses of the target, respectively, whereas *f*_*e*1_ and *f*_*e*2_ denoted the expected numbers of hits and misses, respectively. When a significance level of *p* = 0.05 was applied, a χ^2^ value of greater than 3.84 was considered to be significant. This is equivalent to 8 hits in 20 trials (accuracy: 40%), or 11 hits in 30 trials (accuracy: 36.7%). If subject obtained significant online accuracy, then he/she was considered to be responsive to auditory startle in the BCI experiment. To assist the CRS-R assessment, we polished up the item score by combining both the BCI result and the CRS-R result as follows. If the patient received a score of 1 for auditory startle in the CRS-R assessment, the CRS-R result was trusted regardless of the BCI output. Conversely, if the patient received a score of 0 for auditory startle in CRS-R but the BCI result showed that the patient was responsive to auditory startle, the score was revised to 1. If negative results were obtained in both assessments, the patient received a score of 0.

## Additional Information

**How to cite this article**: Xiao, J. *et al*. An Auditory BCI System for Assisting CRS-R Behavioral Assessment in Patients with Disorders of Consciousness. *Sci. Rep.* **6**, 32917; doi: 10.1038/srep32917 (2016).

## Figures and Tables

**Table 1:** BCI Online and CRS-R Results for DOC Patients.

Patient	Trials	Hits	BCI Online Accuracy (%)	CRS_R Assessment	BCI Assessment
Patient 1	20	15	**75**	1	1
Patient 2	20	11	**55**	1	1
Patient 3	20	15	**75**	1	1
Patient 4	20 (20)	13 (15)	**65** (**75**)	0 (1)	1(1)
Patient 5	20	0	0	0	0
Patient 6	20	8	**40**	1	1
Patient 7	20	6	30	0	0
Patient 8	20	19	**95**	1	1
Patient 9	20	20	**100**	1	1
Patient 10	20	12	**60**	1	1
Patient 11	20 (20)	12 (18)	**60** (**90**)	1 (1)	1(1)
Patient 12	20	12	**60**	0	1
Patient 13	20	14	**70**	0	1
Patient 14	20	9	**45**	1	1
Patient 15	20	17	**85**	1	1
Patient 16	20	12	**60**	1	1
Patient 17	20	12	**60**	1	1
Patient 18	20	19	**95**	1	1
Patient 19	20	15	**75**	1	1

Note: In the column CRS-R assessment, a score of 1 or 0 implies that the behavioral response for auditory startle was detected or not detected, respectively. In the column of BCI assessment, a score of 1 or 0 implies that the auditory startle was detected or not detected, respectively, by our BCI system. The results of the second BCI and CRS-R behavioral measurements were included in the brackets for patients 4 and 11. The accuracies higher than the significance level (40%, *p* = 0.05, χ^2^ test) are highlighted in bold.

**Table 2:** Mean Amplitudes and Peak Latencies of Averaged MMN (FCz) and P300 (Cz) for Healthy, Responsive, Nonresponsive, and Inconsistent groups.

	Healthy Subjects (5)	Patients (19)		
		Responsive group(14)	Nonresponsive group(2)	Inconsistent group(3)
MMN Amplitude (μV)	−3.9 ± 2.2	−2.1 ± 2.2	—	−0.98 ± 0.5 (2)
MMN Latency (ms)	310 ± 5	360 ± 24	—	330 ± 10 (2)
P300 Amplitude (μV)	3.5 ± 1.46	3.0 ± 2.67	—	2.6 ± 0.66 (3)
P300 Latency (ms)	390 ± 10	480 ± 50	—	470 ± 30 (3)

Note: The number of samples for each group is shown in brackets. In the nonresponsive group, including patients 5 and 7, the unambiguous MMN and P300 were not observed. In the inconsistent group including patients 4, 12 and 13, MMN was absent in patient 13; the number of samples who had MMN responses was thus 2.

**Table 3:** Summary of Patients’ Clinical States.

Patients Index	Age	Clinical Diagnosis	Etiology	Time Since Onset (months)	CRS-R Score (auditory-visual-motor-oromotor-communication-arousal)
Patient 1	42	VS	NTBI	4	8 (1-0-4-1-0-2)
Patient 2	28	VS	NTBI	3	6 (1-0-2-1-0-2)
Patient3	54	VS	NTBI	3	7 (1-1-2-1-0-2)
Patient4	40	VS	TBI	6	5 (0-0-2-1-0-2)
		MCS		6	9 (1-1-4-1-0-2)*
Patient 5	47	VS	NTBI	2	8 (0-3-2-1-0-2)
Patient 6	68	VS	NTBI	6	7 (1-1-2-1-0-2)
Patient 7	43	VS	NTBI	3	3 (0-0-1-0-0-2)
Patient 8	66	VS	TBI	1	5 (1-0-1-1-0-2)
Patient 9	28	VS	TBI	3	6 (1-1-1-1-0-2)
Patient 10	16	VS	TBI	1	7 (1-1-2-1-0-2)
Patient 11	35	VS	TBI	6	6 (1-0-2-1-0-2)
		MCS		6.5	11 (1-0-6-1-1-2)*
Patient 12	45	VS	TBI	1	4 (0-0-1-1-0-2)
Patient 13	46	VS	NTBI	1	4 (0-0-1-1-0-2)
Patient 14	72	VS	NTBI	2	4 (1-0-1-0-0-2)
Patient 15	46	MCS	NTBI	3	11 (2-3-3-1-0-2)
Patient 16	63	MCS	NTBI	1	8 (1-1-3-1-0-2)
Patient 17	64	MCS	TBI	2	10 (2-3-2-1-0-2)
Patient 18	57	MCS	NTBI	2	9 (2-2-2-1-0-2)
Patient 19	33	EMCS	NTBI	1	20 (4-4-6-1-2-3)

Note: The CRS-R consists of 6 subscales, i.e., auditory-visual-motor-oromotor- communication-arousal function, and their highest scores are 4, 5, 6, 3, 2 and 3. The total score for the CRS-R ranges from 0 (coma) to 23 (emergence from MCS). TBI: Traumatic Brain Injury; NTBI: Non-Traumatic Brain Injury. The clinical diagnosis and the scores for each patient were based on the CRS-R administered prior to each EEG recording session. Asterisks indicate that the scores were obtained in a second session, for patients 4 and 11.
